# Out-of-home mobility enhancement by a physiotherapist-led motivational counselling intervention among rural community-dwelling older adults 75+: the MOBILE RCT

**DOI:** 10.1186/s12877-026-07244-w

**Published:** 2026-03-05

**Authors:** Christine Haeger, Sandra A. Mümken, Robert P. Spang, Max Brauer, Julie L. O’Sullivan, Sonia Lech, Qian-Li Xue, Martin Stockburger, Jan Keller, Jan-Niklas Voigt-Antons, Paul Gellert

**Affiliations:** 1 Institute of Medical Sociology and Rehabilitation Science, Charité – Universitätsmedizin Berlin, corporate member of Freie Universität Berlin, Humboldt Universität zu Berlin, Berlin, Germany; 2 Quality and Usability Lab, Technische Universität Berlin, Berlin, Germany; 3 Usability Lab, Bauhaus-Universität Weimar, Weimar, Germany; 4 German Centre for Mental Health (DZPG) partner site Berlin, Potsdam, Germany; 5 Department of Psychiatry and Neuroscience, corporate member of Freie Universität Berlin and Humboldt, Charité – Universitätsmedizin Berlin, Universität zu Berlin, Berlin, Germany; 6 Johns Hopkins University Center on Aging and Health, Baltimore, MD USA; 7 Havelland Kliniken Unternehmensgruppe, Berlin, Germany; 8 Department of Education and Psychology, Freie Universität Berlin, Berlin, Germany; 9 Department of Psychology, Heidelberg University, Heidelberg, Germany; 10 Immersive Reality Lab, University of Applied Science Hamm-Lippstadt, Hamm, Germany

**Keywords:** Life-space mobility, Clinical trial, Goal-setting, Home-based

## Abstract

**Background:**

Out-of-home mobility is crucial for maintaining autonomy and participation in older adults and plays a key role in preventing cognitive decline, social isolation, and frailty. However, there is a notable scarcity of interventional research aimed at promoting out-of-home mobility, particularly in rural areas where environmental barriers to mobility are pronounced and older adults are at risk of limited mobility. This study aimed to evaluate the effectiveness of a physiotherapist-led motivational counselling intervention designed to enhance out-of-home mobility in older adults living in rural communities.

**Methods:**

A randomized controlled trial was conducted among community-dwelling older adults aged 75 and above, residing in a rural region of Germany. Participants in the intervention group received motivational counselling focusing on goal setting, social network activation, and the use of regional resources, while the control group received an informational leaflet. The primary outcome, out-of-home mobility (time out of home), was assessed using GPS tracking over seven consecutive days at T_0_ (baseline), and at T_1_ and T_2_ (4 and 12-week follow-ups). Secondary outcomes included GPS-derived convex hull, self-reported life-space mobility, physical activity, depressive symptoms, health status, as well as frailty. Mixed-effects models were employed and effect sizes were estimated using Cohen’s d.

**Results:**

A total of 212 participants (mean age 81.54 ± 4.0 years) were included, with 108 participants in the intervention group. Both groups showed an increase in out-of-home mobility over time. However, no significant intervention effects were observed at T_1_ and T_2_ for either the primary or secondary outcomes (all *p* > .05). Subgroup analyses revealed significant interaction effects for women, pre-frail/frail individuals, participants aged 81 and older, and those residing in the most rural municipalities (≤ 15,000 inhabitants) for physical activity, health status and life-space mobility.

**Conclusions:**

Both, the intervention and control groups exhibited an increase in out-of-home mobility. However, no differential intervention effect was observed, possibly reflecting the already active baseline characteristics of the sample. Subgroup analyses identified significant interaction effects among particular subgroups, pointing towards a greater necessity for targeted interventions in future investigations. Further research is warranted to explore how to effectively improve mobility in older rural populations.

**Trial registration:**

The trial was prospectively registered at the DRKS registry on 5 May 2021 (Deutsches Register Klinischer Studien) with the study identity number DRKS00025230. Ethical approval was given by the Ethics Committee of Charité – Universitätsmedizin Berlin (EA1/052/20).

**Supplementary Information:**

The online version contains supplementary material available at 10.1186/s12877-026-07244-w.

## Background

Out-of-home mobility is important for older adults to maintain a self-determined life, allowing them to participate in social and physical activities [1, 2]. Restrictions in out-of-home mobility are associated with various adverse health outcomes such as the onset of frailty, cognitive decline, disability, and depression [3–5]. Especially in rural areas, where residents experience mobility barriers like reduced access to services and activities, poorer infrastructure or lower walkability, it is observed that older adults face greater risk of reduced out-of-home mobility increasing the likelihood of mentioned adverse health outcomes [6].

Out-of-home mobility has been defined as any active or passive movement through diverse outdoor environments using the life-space concept [7]. Traditional assessments of life-space and out-of-home mobility include self-reported activity diaries or questionnaires such as the widely used Life-Space Assessment (LSA) [8]. More recently, Geographic Positioning System (GPS) sensors have been applied to objectively capture out-of-home mobility behaviour and permit the precise assessment of its temporal and spatial aspects [9, 10].

There are several theories that seek to explain the factors influencing out-of-home mobility in old age. One prominent example is the Life-Space Constriction Framework by Xue et al. [11], which proposes personal (e.g., compensatory strategies and assistive devices), social (e.g., family ties and social networks), and environmental factors (e.g., area deprivation, walkability) contributing to life-space constriction, which, in turn, is associated with distal outcomes including loss of capacity, frailty, and mortality [11]. Hence, targeting these three dimensions in interventions may improve out-of-home mobility and support frailty prevention.

Although there is a substantial body of literature drawing on observational evidence of factors associated with out-of-home mobility, empirical evidence from intervention studies remains scarce. A recent systematic review summarized interventions aiming to increase life-space mobility in older adults, identifying 16 randomized controlled trials (RCT) and reported mixed results [12] indicating a need for further research. An additional recent RCT integrating a counselling component to standard physical training found an effect on GPS-measured indicators [13]. Among these trials, only one focused on rural areas [14], two used GPS-derived measure of life-space mobility [13, 15] and four incorporated counselling components with mixed results [13, 14, 16, 17]. To our knowledge, no studies have examined counselling interventions and their effect on out-of-home mobility in rural older adults. Older adults living in rural areas experience higher unmet out-of-home mobility needs than those living in urban areas [18], and especially after giving up driving, challenges for out-of-home mobility increase [19]. On the other hand, rural areas provide particular resources for out-of-home mobility such as larger social networks [20] and the proximity to green natural environments is associated with better physical health [21]. As out-of-home mobility facilitates aging in place [22] and a good quality of life [23], there is a need for interventions that promote individualized strategies to maintain out-of-home mobility, taking into account the specific barriers and resources of rural environments [24]. By addressing personal, social, and environmental resources [11], the aim of the MOBILE trial (Mobility in Old age By Integrating health care and personal network resources in older adults Living in rural arEas) was to test the effectiveness of a physiotherapist-led home visit motivational counselling intervention on enhancing out-of-home mobility in older adults 75 years and older living in a rural area in Germany. Home visits have shown to be effective in lifestyle changes [25]. Counselling is an interactive process drawing on motivational and self-regulatory concepts [26, 27].

Our primary hypothesis was as follows: Compared to the control group, participants in the intervention significantly increase their TOH at week 4 and week 12. Further, we assumed improvements among secondary outcomes (i.e., GPS-derived convex hull, self-reported life-space mobility, physical activity, depressive symptoms, physical and mental functioning, as well as frailty) between the intervention and control group at follow-ups. Finally, we aimed to investigate whether our intervention is successful in increasing out-of-home mobility equally for subgroups (i.e., by gender, frailty, age, and population size of the municipality).

## Methods

MOBILE was a RCT to enhance out-of-home mobility among older adults aged 75 + living in the Havelland region, a rural area in Germany. In this study, a “rural area” is defined according to the Eurostat Degree of Urbanisation (DEGURBA) classification for NUTS‑3 regions, which categorizes regions based on population density and settlement patterns, considering those in which more than 50% of the population resides in rural grid cells as predominantly rural [28]. The Havelland region is a rural area located west of Berlin and comprises 26 municipalities. Three of these are rural small towns (i.e., Nauen, Rathenow, Falkensee) with more than 15,000 inhabitants, while the remaining municipalities are predominantly rural, consisting of villages and smaller settlements with vast areas of woods and farming land, surrounding lakeside and a population density of 98 person per km^2^. We adopted a community-oriented approach, fostering collaborations with local stakeholders such as municipal politics, healthcare institutions, senior advisory bodies, and preventive services [29]. The study was funded by the German Federal Ministry of Education and Research (ID: 01GY1803) and was pre-registered at the German Clinical Trial Register (DRKS00025230, 2021/05/04). The study was approved by the local ethics committee of Charité – Universitätsmedizin Berlin, Germany (reference number EA1/052/20) and conducted in accordance with the Declaration of Helsinki. Details of the study design were described in the study protocol [29].

Data was collected as a computer-assisted face-to-face interview at three time points (T_0_ = baseline, T_1_ = 4 weeks after baseline, T_2_ = 12 weeks after baseline). Due to the COVID-19 pandemic, home-based interviews were switched to telephone interviews in December 2021 for 10 participants. This adaptation to the pandemic situation was described in the study protocol and did not impair the quality of the data. To further minimize the risk of infection, all contacts were in compliance with hygiene regulation of the federal state Berlin and the Charité – Universitätsmedizin Berlin including full vaccination, self-testing, wearing FFP2-masks, ventilation and hand sanitizing. However, those restrictions did not affect the ongoing of the study. Adverse events during or following the home visits were monitored and recorded.

All participants were asked to carry a GPS study device consistently for seven consecutive days and simultaneously maintain an activity diary during each measurement period (T_0_, T_1_, and T_2_) to document their out-of-home mobility. We allowed for a longer tracking duration (up to 14 days for each measuring point) if participants wished to do so. For the GPS data collection, an open-source smartphone application “GPS.Rec.2.0” was developed [30] and installed on basic smartphones (ZTE Blade A5) provided only for each measurement period and returned afterward using a postage-prepaid return envelope, which allowed direct data transfer and ensured continuous data quality. It is worth noting that this application works well in rural areas as the battery lasts 48 h and it works without any internet connection. For further technical details on development, accuracy and usability of the smartphone application see the MOBILE technical paper [30].

### Participants

Participants from the Havelland region were mainly recruited through population-based postal invitation letters (70%) stratified by age. Addresses were obtained upon formal request from the responsible local registration offices for the purpose of study recruitment in the public interest. Within the region’s 26 municipalities, individuals were randomly selected based on postal codes, with particular emphasis on municipalities where no participants had yet been enrolled, to improve geographic representation and the inclusion of hard-to-reach individuals. Further, other recruitment strategies included advertisements in local newspaper and announcements in (activity) groups. Inclusion criteria were age ≥ 75 years, living in the Havelland region, community-dwelling, having sufficient mobility (i.e., being able to move autonomously or with walking aids and/or little help of others), ability to give informed consent, and were willing to participate. Eligibility was assessed via a standardized telephone screening focusing on functional limitations rather than specific diagnoses. Exclusion criteria were severe cognitive impairments (defined as an apparent inability to understand study information during screening), residence in long-term care facilities, severe psychological or emotional impairments (self-reported conditions substantially limiting safe participation), severe events impairing mobility within the past four weeks (e.g., self-reported surgery, acute injury, or other health events clearly affecting mobility), or insufficient understanding of the German language.

### Randomization

We used a stratified randomization technique based on the criteria age (> 80 vs. 75–80 years) and population size of the municipality (> 15,000 inhabitants vs. ≤ 15,000 inhabitants) with a 1:1 allocation to ensure balanced participant characteristics in both groups (couples were assigned to the same group to avoid contamination). Randomization was conducted using fixed blocks of four, six, and eight to maintain balance within each stratum. Allocation was performed by study personnel after enrolment using the R package “blockrand” (R version 4.0.4) and was concealed at baseline through a study-specific Microsoft Access database, with group assignment revealed only after completion and entry of the baseline assessment by the study nurse. Due to the nature of the intervention, blinding and allocation concealment could not be maintained after this point.

### Intervention

**Fig. 1:**
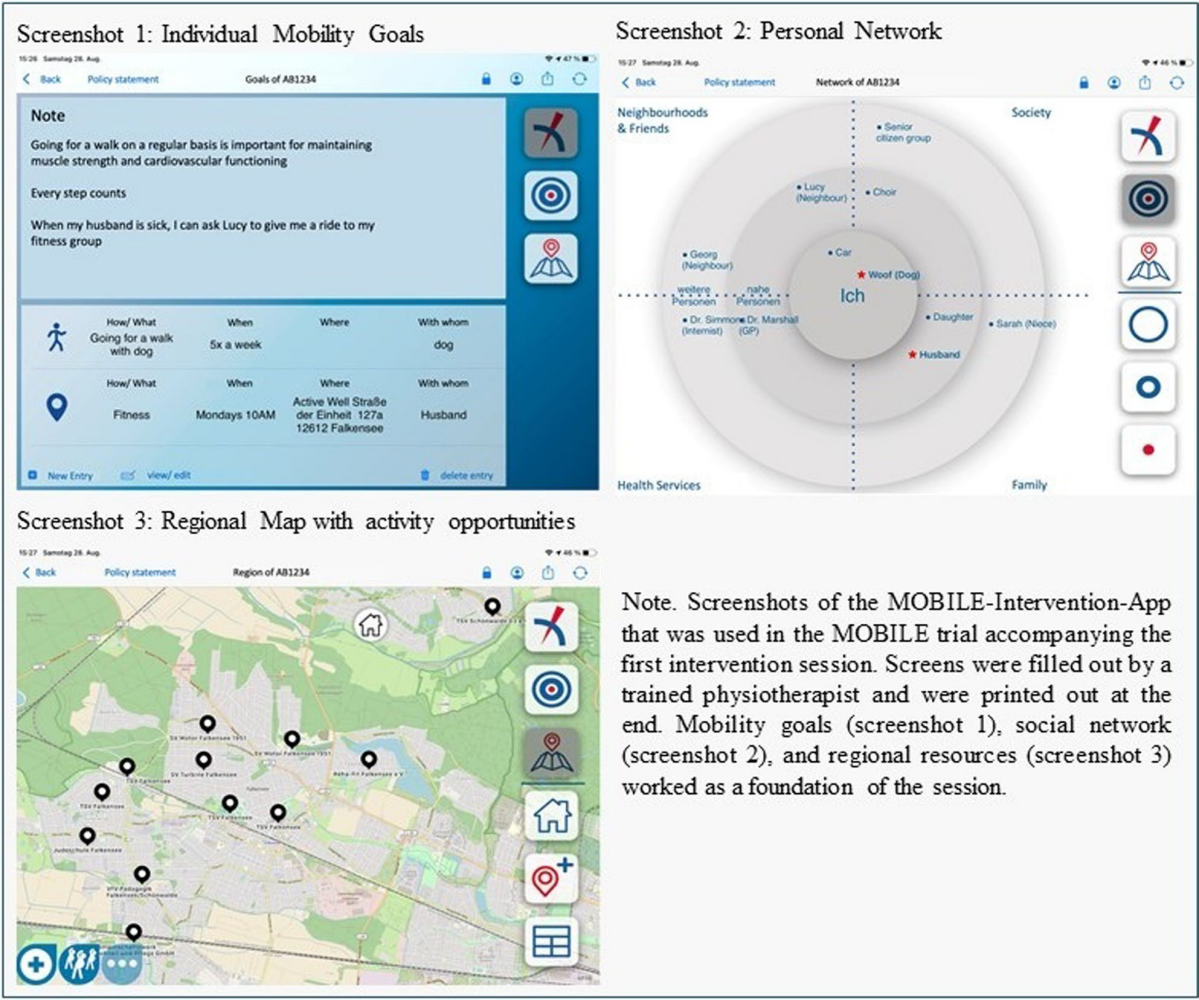
Outline of the “MOBILE-Intervention-App” that was developed for intervention delivery. Figure adapted from the study protocol [29]

The motivational counselling intervention aimed to increase out-of-home-mobility by targeting personal, social, and environmental resources and was performed by trained physiotherapists. A standardized training was provided based on a theory-led study manual. Details on the intervention are described in the study protocol [29] and summarized here as well as in the Template for Intervention Description and Replication (TIDieR) [31], see supplement (eTable 1). In brief, participants of the intervention group received a 2-hour face-to-face motivational home visit two weeks after the baseline assessment supported by the “MOBILE-Intervention-App” (see Fig. 1) followed by two booster telephone calls. The main intention of the first home-visit session was to set clearly defined personal out-of-home mobility goals and visualize them on screen. We asked our participants to set two goals: One goal with the intention to be incorporated in the daily/weekly mobility routine for sustainable changes (like going for a walk every Monday) and one with a special destination (like going to the museum this Saturday). The app served as a structuring element with three components: (1) personal out-of-home mobility goals (i.e., addressing personal resources); (2) a social network map that is filled out within the session (i.e., addressing social resources); and (3) a map (OpenStreetMap^®^) with predefined locations for engaging in certain activities (i.e., addressing regional resources). The home-visit and the booster telephone calls were based on established behavior change techniques [32], especially focusing on goal setting, action planning, habit formation (as part of personal resources), and social support (as social resources). Two follow-up telephone calls (i.e., four and eight weeks after the first session) focused on goal adjustment; that is, participants had the opportunity to review their goals and adapt or alter those. Special focus was thereby set on resources and barriers for reaching the goals (e.g., asking the neighbor to join the walk if the usual companion is not available). Screenshots (i.e. goals and social network) were printed out at the end of the first session for the participants to keep and worked as a starting point for the telephone booster calls.

The control group received an eight-page booklet called “brochure for an active everyday life” containing general health information in layman’s terms (e.g., why activity boosts the immune system, why mobility is especially important for older adults) as well as area-specific information (e.g., a list of places to engage). Participants could fill out out-of-home mobility goals on the last page and the booklet was sent to their homes two weeks after the baseline assessment to align the study timeline with that of the intervention group.

### Primary outcome

Out-of-home mobility as the primary outcome was measured using the GPS-aggregated variable “time out-of-home” (TOH) at T_0_, T_1_, and T_2_. The variable was based on the GPS data implying the time spent outside of a 50 m home buffer. We chose TOH as primary outcome, as TOH is one of the most commonly used GPS-derived mobility indicators in behavioral GPS studies [9, 10] and as it suits the aim of our intervention as it captures all tailored out-of-home mobility goals independent from geographic distance outside the home buffer. The TOH variable is aggregated, applying an own developed Stop & Go classifier [33, 34] that differentiates between a stop and a trip. Accuracy analyses show a correct identification in 96.79% of the cases and a F1-score of 0.966 both indicating excellence performance [35]. Further, we followed the recommendations of Fillekes et al. [9] and defined days with a minimum recording of 8 h as valid study days. We then included all valid study days in our analyses. TOH is measured in minutes and can theoretically range from 0 to 1440 min for a given day; we calculated TOH for every study day. Days were defined from 3:00:00 AM to 2:59:59 AM to account activities expanding midnight to the previous day, as recommended in the literature [9]. For further details on GPS data collection and analysis during the MOBILE trial, see our technical paper [35].

### Secondary outcomes

Secondary outcomes included GPS-measured life-space mobility as well as self-reported life-space mobility, physical activity, depressive symptoms, health status, and frailty.

We defined *GPS-derived life-space mobility* as the *area of convex hull* in m^2^ (CHull). The area of convex hull encloses all daily GPS-measured data points within the smallest convex polygon and therefore reflects the extent of the daily visited movement area [9].

*Self-reported life-space mobility* was assessed using the composite score (LS-C) of the Life-Space Assessment - German version, LSA-D [36]. The questionnaire covers six life-space levels (i.e., bedroom, apartment, near living environment, neighborhood, city, and beyond), the frequency of attendance of the six life-space levels and need of assistance (devices and/or from persons) over the last four weeks. The LS-C combines components of attained life-space levels, frequency, and need of assistance and ranges from 0 to 120 with higher scores indicating greater mobility.

*Physical activity* was measured with the short version of the International Physical Activity Questionnaire (IPAQ) [37] which has been validated for older adults [38]. Participants were asked about the number of days and duration regarding their performance of physical activities consisting of (a) vigorous intensity, (b) moderate intensity, or (c) walking across the past week. Examples of activities were given in order to increase comprehension. MET-minutes/week were calculated (walking = 3.3 METs, moderate = 4.0 METs, vigorous = 8.0 METs), and participants were classified into low (not meeting criteria for moderate or high activity), moderate (≥ 600 MET-min/week or meeting frequency/duration thresholds), and high activity (≥ 1500 MET-min/week vigorous activity or ≥ 3000 MET-min/week total activity), in addition to a continuous total MET-minutes/week score [37].

*Depressive symptoms* were measured using the Geriatric Depression Scale ( Residential) (GDS-12R) [39]. The scale consists of 12 questions (example: “Are you basically satisfied with your life?”) that can be answered with yes or no. Final scores range from 0 to 12 with higher scores indicating more depressive symptoms (a score of 4 or more indicates the presence of clinically relevant depressive symptoms).

For *health status*, we used the Short-Form Health Survey (SF-12) [40] that measures health-related quality of life. The SF-12 is a short version of the predecessor SF-36 with high validity and reliability. A physical (PCS12) and a mental (MCS12) subscale can be distinguished with higher scores indicating higher health-related quality of life (range 0-100).

*Frailty* was assessed using the frailty phenotype proposed by Fried et al. [41] that encompasses five domains (i.e., exhaustion, weight loss, low activity, slow walk, grip strengths) for classifying the participants as either non frail, pre-frail or frail, and further dichotomized for statistical purposes in 0 = non-frail and1 = pre-frail or frail. Questionnaires, a gauged hand grip dynamometer, and a stopwatch were used for examination.

### Additional baseline measures

We obtained several additional baseline measures (T_0_) including age, gender (self-reported: men, women, diverse), educational level (based on highest academic or professional degree and graded according to the International Standard Classification of Education ISCED [42]), level of care (long-term care levels ranging from 1 (lowest care dependency) to 5 (highest care dependency) based on the German Social Code, Book XI), living arrangements (living alone, living with a partner, living with a relative or other person, assisted living), physical performance (DeMorton Mobility Index [43]), hand grip strength (measured with a gauged hand grip Dynamometer Sahean SH5001), history of falls, utilization of assistive devices (adapted from [8]), cognition (using the short screening test Mini-Cog [44]), four COVID-19-specific items (i.e., infection and vaccination history, level of fear, and impact on personal life), and living surroundings (i.e., type of building, floor, steps in front or inside building, and quality of footpath). All these additional baseline measurements are included as covariates in the analyses.

### Sample size calculation

Sample size was estimated using an open-source power calculator [45] based on standard formulas [46] by considering an increase in time spent TOH of 15% after three months (compare: Portegijs et al. [4]) within the intervention group (1:1 ratio, power 1 – beta = 95%, alpha [two-sided] = 5%, dropout = 15%). Based on these presumptions, a total sample of *N* = 254 (*N* = 127 in each condition) is needed.

### Statistical analysis

Descriptive data are presented in absolute and relative frequencies for categorical data and as mean and standard deviation for continuous variables. Differences between participants who discontinued the study were determined with unpaired t-tests and chi square tests. Intervention adherence was calculated based on the proportion that took part in the scheduled follow-up telephone calls.

Generalized linear effect models (GLMM) and linear mixed-effect models (LMM) with time points nested in participants were performed using an intention-to-treat approach [47]. We used GLMM assuming a Poisson distribution for the primary outcome and GPS-derived life-space variable that were both identified as a count variable (i.e., daily minutes of TOH) and LMMs for all other outcomes. We specified individual days as level 1, nested within the person at level 2 to account for the hierarchical data structure. We included all baseline measures as covariates, as well as couples’ IDs (dummy coded) and a counter variable to account for daily variance in the GPS-based outcomes and for potential intra-couple dependencies. The inclusion of couples’ IDs was necessary because partners may share similar behavioral patterns (e.g., TOH), which can lead to correlated observations and reduced independence of the data. By including these couple ID dummy variables, we adjusted for shared variance within couples and reduce residual variance attributable to couple-specific effects. LMM models were fit using restricted maximum likelihood (REML) and GLMM models using maximum likelihood (Laplace Approximation) procedures. Continuous variables were grand mean centered and dichotomous variables were coded with 0 and 1. A group × time interaction term was added to determine the effect of the intervention across measurement points. Effect sizes were calculated according to Cohen’s d formula [48]. To account for multiplicity, all p-values were adjusted using Hochberg-method [49]. Subgroup analyses were performed for men and women. Missing GPS values within a tracked day, that were due to commonly occurring GPS signal losses, were interpreted based on the time between two subsequent samples and their spatial distance [34]. Days with no GPS signal (e.g., due to either technical issue or forgotten device) were excluded from analyses as suggested in the literature [9, 50] and implied in a comparable trial [13]. Thus, no GPS data imputation was performed and missing values were handled with maximum likelihood estimations [51]. Subgroup analyses encompass analyses of primary and secondary outcomes stratified by gender, frailty, age, and population size of the municipalities. The groups for age (> 80 years vs. 75–80 years) and population size of the municipalities (> 15,000 inhabitants vs. ≤ 15,000 inhabitants)were based on pre-specified randomization and are stated in the study protocol [29], whereas analyses of subgroups based on gender (men vs. women [any participant was self-identifying as divers]) and frailty (pre-frail or frail vs. robust) were post-hoc instigated. Significance level of 0.05, and confidence interval (CI) of 95% were used and statistical analyses were performed using R version 4.3.0 and SPSS Version 29 for Windows (IBM, Cary., Ind.).

## Results

**Fig. 2:**
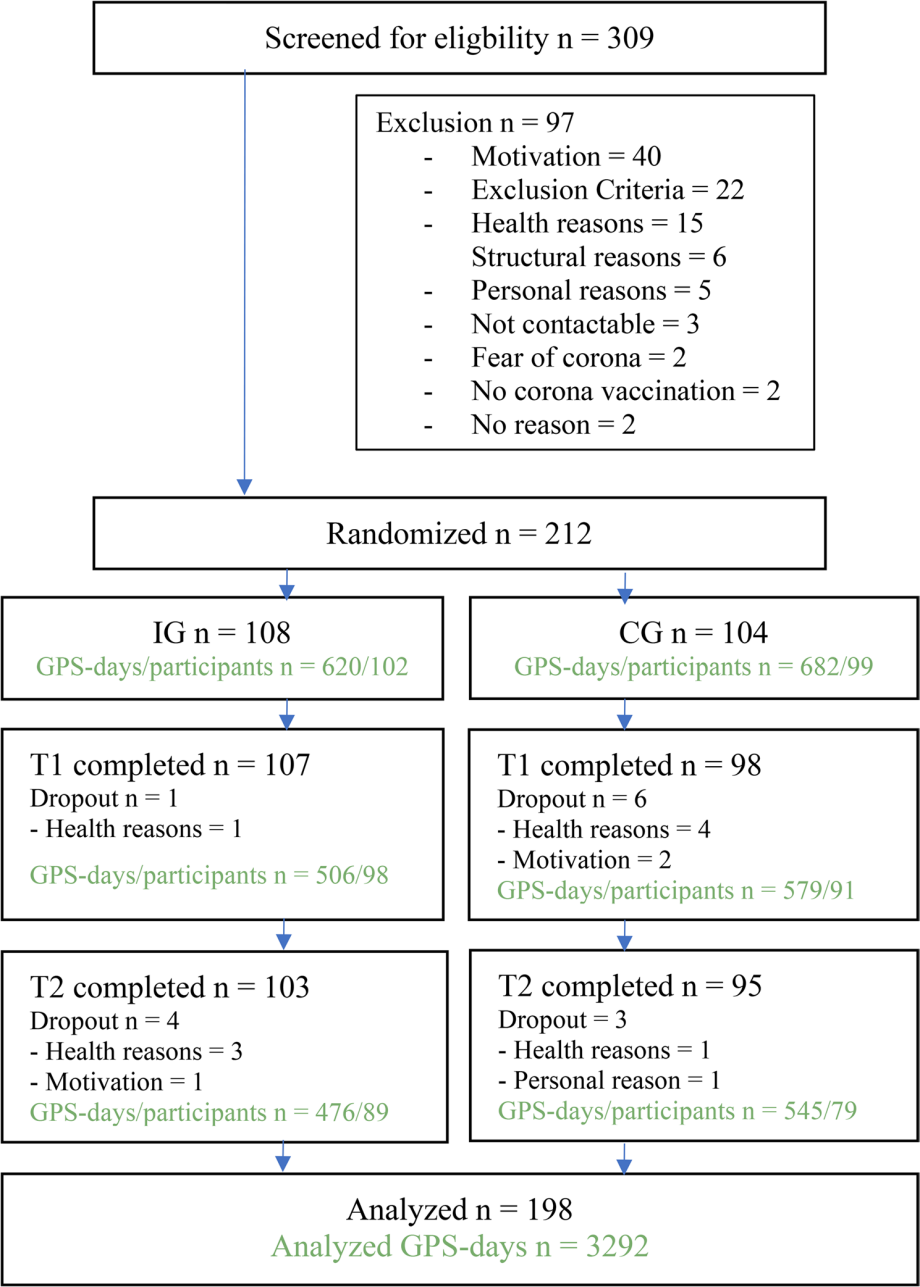
Flowchart of the MOBILE trial. Legend: IG = intervention group, CG = control group, green numbers indicate valid GPS-days in relation to number of participants

From June 2021 to October 2022, we screened 309 community-dwelling older adults aged 75 years or older from the Havelland region, 212 of them were eligible and willing to participate (recruitment rate 69.0%). Main causes for non-participation were lack of motivation (*n* = 40), meeting exclusion criteria (*n* = 22), and health reasons (*n* = 15) (see Fig. 2).

Randomization took place after the baseline measurement, yielding 108 participants in the intervention group and 104 participants in the control group (Fig. 2) with comparable baseline data (Table 1). The age of the participants ranged from 75 to 94 years with a mean age of 81.5 years, 56.1% were women. 55.0% were classified as pre-frail and 10.0% as frail. The average TOH was 319.3 min per day (SD: 196.3).

**Table 1:** Baseline characteristics of intervention and control group of MOBILE trial (*N* = 212)

Categorical variables	IG (*n* = 108) *n* (%)	CG (*n* = 104) *n* (%)
Gender, women	60 (55.6)	59 (56.7)
Population size of municipality, ≤ 15,000 inhabitants	59 (54.6)	50 (48.1)
Education		
Basic	2 (1.9)	3 (2.8)
Vocational	47 (45.2)	38 (35.2)
Degree	55 (52.9)	67 (62.0)
Level of care		
No level of care	88 (18.5)	80 (76.9)
Level of care 1	5 (4.6)	8 (7.7)
Level of care 2	14 (13.0)	13 (12.5)
Level of care 3	1 (0.9)	3 (2.9)
Living arrangements		
Living alone	41 (38.0)	44 (42.3)
Not living alone	67 (62.0)	60 (57.7)
Use of assistive devices		
Walking aid	24 (22.2)	28 (26.9)
Hearing aid	36 (33.3)	39 (37.5)
Visual aid	107 (99.1)	101 (97.4)
Smartphone	95 (88.0)	88 (84.6)
Frailty		
Robust	43 (39.8)	30 (29.7)
Pre-frail	55 (50.9)	60 (59.4)
Frail	10 (9.3)	11 (10.9)
Physical activity (IPAQ)		
Low	8 (7.4)	12 (11.5)
Moderate	18 (16.7)	16 (15.4)
High	82 (75.9)	76 (73.1)

Continuous variables	Mean (SD)	Mean (SD)
Age	81.18 (3.76)	81.92 (4.36)
Mobility		
TOH (*n* = 201)	316.3 (204.5)	322.3 (188.5)
CHull (*n* = 201)	34.4 (59.5)	50.4 (179.1)
LS-C	79.6 (21.3)	75.5 (18.9)
Physical activity (IPAQ)		
MET-minutes/week	5072.3 (2984.1)	4860.5 (2913.3)
Depressive symptoms		
GDS-12R	0.9 (1.4)	1.1 (1.7)
Health status SF-12		
PCS12	43.1 (11.6)	44.7 (10.8)
MCS12	58.8 (4.8)	57.8 (5.9)

Values are presented frequencies for categorical and in means and standard deviation (SD) for continuous variables

*IG* Intervention Group, *CG* Control Group, *SD* Standard Deviation, *TOH* Time-out-of-home, *CHull* Convex Hull (= GPS-Based Life-Space parameter), *LS-C* Composite Score of the Life-Space Assessment), *IPAQ* International Physical Activity Questionnaire, *MET* metabolic equivalent of an activity, *GDS-12R* Geriatric Depression Scale (Residential), *SF-12* Short Form Health Survey, *PCS12* Physical Component Summary Scale of SF-12, *MCS* Mental Component Summary Scale of SF-12

We observed a higher retention rate in the intervention group. Five participants (4.6%) in this group discontinued mainly due to health reasons, whereas in the control group nine participants discontinued (8.6%) as shown in Fig. 2. 198 participants were included in the final analyses with a total of 3,292 valid GPS-days for T_0_-T_2_ (mean 16.6 days/participant). The average number of participants with valid GPS-days varied between time points (T_0_: 201 participants, T_1_: 189 participants, T_2_: 168 participants, see Fig. 2). The median number of valid GPS days was 7 at T_0_ and T_2_ and 6 at T_1_ (across both treatment arms), with an overall GPS data completeness regarding participants with valid GPS data between 72 and 90% depending on time point and study arm. In 5% of cases, the tracking period exceeded 7 days (8 days in most instances, with one case in the control arm reaching 14 days). Comparisons between study completers and drop-outs showed that in terms of sociodemographic characteristics (i.e., age, gender, education) there was a significant difference in terms of education (*p* < .001), but no differences were found regarding age or gender (see Supplement: eTable2).

### Primary and secondary outcomes

Intervention effects (time × group) for primary and secondary outcomes are presented in Table 2. The adjusted estimated marginal means (EMMs) of TOH at baseline were 252 (95% CI: 199–320) min/day for the intervention and 251 (95% CI: 200–300) min/day for the control condition. While we observed significant increases in adjusted EMMs of TOH from baseline to T_1_ (ß 0.26, *p* < .001), we found no significant intervention effect at either of the follow-up measurements (T_1,_*p* = .923 (time × group), *d* = 0.06, T_2_, *p* = .923 (time × group), *d* = 0.04). There was no further increase in TOH from T_1_ to T_2_, but the TOH level was remaining significantly higher compared with the baseline in both groups. Regarding secondary outcomes (i.e., CHull, LS-C, IPAQ, GDS-12R, PCS12, MCS12, frailty status), no intervention effects were found (all *p* > .05). All results for the primary and secondary outcomes are presented in Table 2.

**Table 2:** Estimated marginal means and effect sizes for primary and secondary outcomes for intervention and control group of the MOBILE trial, adjusted for baseline values

Outcome	T_0_ (Baseline)		T_1_ (4-week follow-up)				T_2_ (12-week follow-up)			
	IG (95% CI)	CG (95% CI)	IG (95% CI)	CG (95% CI)	*P*	Cohen`s d (95% CI)	IG (95% CI)	CG (95% CI)	*P*	Cohen`s d (95% CI)
TOH in min/week	252 (199–320)	251 (200–300)	316 (241–415)^1^	338 (262–436)^1^	.923	0.06 (-0.18–0.31)	316 (242–413)^1^	305 (237–392)^1^	.923	0.04 (-0.28–0.20)
CHull in m^2^	2.62 (1.22–5.64)	2.24 (1.08–4.66)	1.46 (0.63–3.40)^1^	1.92 (0.87–4.27)^1^	.923	0.27 (-0.50–1.02)	1.60 (0.70–3.65)^1^	2.03 (0.93–4.42)^1^	.923	0.23 (-0.46–0.94)
LS-C (0-120)	73.3 (67.7–78.9)	71.3 (66.0–76.6)	71.4 (65.7–77.1)	66.3 (60.9–71.6)	.923	0.31 (0.17–0.80)	68.0 (62.2–73.8)	69.4 (64.0–74.9)	.923	0.01 (-0.50–0.48)
IPAQ in MET-min/week	3654 (3141–4398)	2697 (2190–3110)	4058 (3351–4746)	2882 (2199–3564)	.354	0.64 (0.25–1.04)	3821 (3112–4530)	2733 (2049–3416)	.923	0.59 (0.21–0.99)
GDS-12R^2^ (0–12)	1.69 (1.20–2.19)	1.58 (1.11–2.05)	-	-	-	-	1.74 (1.24–2.24)	1.67 (1.20–2.14)	.923	0.07 (-0.45–0.33)
PCS12 (0-100)	41.0 (38.2–43.8)	39.0 (36.0–41.9)	39.0 (35.9–42.1)	38.8 (35. – 41.7)	.923	0.03 (-0.47–0.40)	38.9 (35.8–41.0)	38.4 (35.4–41.3)	.923	0.08 (-0.52–0.35)
MCS12 (0-100)	57.6 (55.8–59.4)	57.2 (55.5–58.9)	57.1 (55.1–59.0)	56.8 (55.0–58.6)	.923	0.07 (-0.49–0.33)	57.2 (55.2–59.1)	57.4 (55.5–59.2)	.923	0.05 (-0.37–0.47)
Frailty^2^ (0–1)	0.79 (0.67–0.91)	0.76 (0.64–0.87)	-	-	-	-	0.79 (0.67– 0.90)	0.76 (0.64–0.87)	.923	0.07 (-0.45–0.30)

*IG* Intervention Group, *CG* Control Group, *CI* Confidence Interval, *TOH* Time-out-of-home, *CHull* Convex Hull, *LS-C* Composite Score of the Life-Space Assessment, *IPAQ* International Physical Activity Questionnaire, *GDS-12R* Geriatric Depression Scale (Residential), *PCS12* Physical Component Summary Score of the Short Form Health Survey (SF-12), *MCS12* Mental Component Summary Score of the Short Form Health Survey (SF-12)

^1^Intervals are back-transformed from the log scale

^2^Were not assessed at T_1_

Group means and differences were estimated with generalized linear mixed models (GLMM) and linear mixed models (LMM). All models are adjusted for particular baseline measures (age, gender, education, physical functioning, hand grip, history of falls, use of assistive devices, mini-cog, COVID-19 specific items, level of care, living arrangements, living surroundings), and results are based on 95% confidence interval. The number of digits displayed is justified by the standard error of the estimates

*P*-values are reported from the interaction terms’ coefficients and adjusted for Hochberg correction

### Explorative subgroup analyses

Additional explorative subgroup analyses were performed for the variables age, gender, frailty status, and population size of municipality. Intervention effects (group × time) were analyzed within each subgroup (for baseline characteristics of subgroups see supplement: eTable 3–6). We found a significant effect for women at T_1_ for LS-C, IPAQ, and MCS12 with higher scores compared to the counterparts (all *p* < .05). All other outcomes for women were non-significant and no significant effects for T_2_ in any of the analyses. For the subgroup “age > 80 years” a significant effect was demonstrated for the IPAQ at T_1_ as well as for the pre-frail and frail subgroup (*p* < .005). For the robust subgroup, a significant effect is shown at T_2_ (*p* < .05). Finally, there were significant effects on the subgroup in most rural areas (≤ 15,000 inhabitants) at T_1_ for the IPAQ (*p <* .05) and PCS12 (*p <* .005). No effects were found at T_2_. Significant intervention effects are visualized in the supplement (see supplement: eFigure 1).

### Adverse events

There was one adverse event recorded, where the participant felt dizzy and weak, and the intervention had to stop preterm. Two participants also felt dizzy during the conduction of the assessments and needed a short break but continued the questionnaire afterwards. Other events or reasons for dropout of participants were not in relation to our study.

## Discussion

### Primary outcome

We found no intervention effect on TOH on either of the follow-up measures. One explanation for this null-finding might a be a ceiling-effect as the participants had high baseline values for TOH, thus, the threshold for achieving detectable improvements was high. Two comparable studies, both with high baseline values, echo our results [13, 15]. It became apparent during our intervention that it is challenging to set goals for participants who were already active and felt no need to engage more. Narrowing the target population down to those with restricted out-of-home mobility could have led to significant intervention effects. Another explanation might be the fact that TOH increased equally in both groups from T_0_ to T_1_ and remained on that level at T_2_. This finding may be indicating that we can improve TOH by drawing attention to it and addressing the resources for achieving it. Further, the GPS tracking might have influenced participant’s behavior in spending more TOH in both groups and the control group could have been motivated by the information brochure as well, where tips were given on how to incorporate more out-of-home mobility in everyday life. Our intervention focused on goal setting components and participants were encouraged to set out-of-home mobility goals that would fit to their capacities and interests; however, we did not ask our participants to set goals that exceed a certain number of minutes spent out-of-home (e.g., 3 h every day). Therefore, performed out-of-home activities encompassing only short durations might have been less recognized. While we chose an intervention that purely focused on counselling, Seinsche et al. suggest in a recent review that multidimensional interventions (e.g., combining counselling components with physical exercise) might be more effective [12]. It is further arguable whether TOH, although state of the art, is the best indicator in detecting changes in out-of-home mobility behavior or if a more fine-grained GPS indicator, capturing more nuanced details in mobility, is needed to be developed.

### Secondary outcomes

While we found no effects on life-space parameters (LS-C and CHull), several previous trials did find significant post-intervention improvements [14, 52–54]. However, these aforementioned trials included specific samples such as hospitalized individuals [52], participants recently discharged from geriatric rehabilitation [53], those with low health literacy [14], and those living in nursing homes [54]. It is highly likely that these groups are far more vulnerable to having decreased life-space mobility and thus interventions for increasing life-space mobility may be more effective. While our investigation did not yield any significant group differences regarding physical activity, two comparable trials reported significant improvements on physical activity [14] and walking time [15]. Crist et al. further report that even though walking time increased, TOH remained the same, indicating that the intervention had an effect on the quality of transportation, an important differentiation that future research should focus on.

No difference was demonstrated for GDS-12R in the present study. This finding may be attributed to the fact that baseline values indicate a mentally stable sample, thus no clinically relevant impact was to be expected – an observation which is consistent with findings from previous trials [16, 54]. Further, the 12-week follow-up period might have been too short to pick up significant changes. No effects were found for health-related quality of life and baseline values were again high in our sample compared to German norm values [55], whereas Crotty et al. (2019) found a small effect after twelve months; albeit in a sample of people with cognitive impairments. However, in our trial as well as in the trial reported by Crotty et al. (2019) retrospective self-reported instruments were used and recall-biases may have occurred. Integrating ecological momentary assessments could be effective in detecting changes in health-related quality of life directly connected to the intervention. Finally, no significant effect was detected on the secondary outcome frailty. This is in line with Fairhall et al. (2012), who however, found subgroup effects when comparing frailer to less frail older adults with stronger effects for those with enhanced frailty. This, again, hints towards a more specific target population for a more effective intervention.

### Subgroup analyses

We performed subgroup analyses for age, gender, frailty status, and population size of the municipalities. These analyses revealed a significant gender difference with women showing post-intervention improvements regarding LS-C, IPAQ, and MCS12. These findings indicate that our intervention was more effective in women compared to men, at least regarding these outcomes. Crist et al. also looked into gender differences but found mixed results [15]. Women showed a greater increase in beyond neighborhood (> 800 m from home) walking at 3 months, while men showed an increase in walking in their nearby living environment (campus domain) at 6 months. Furthermore, when conducting separate analyses for pre-frail and frail participants vs. participants without frailty, we found differential effects. Those who were pre-frail and frail showed increased IPAQ at T_1_, whereas those without frailty showed an increase at T_2_. Hence, we reached both subgroups for targeting frailty; however, the time-lagged post-intervention effect seemed to differ. This could be a hint that interventions targeting pre-frail and frail older adults need to include more frequent counselling interventions to maintain effects over longer time periods. Fairhall et al. [56], who conducted a complex intervention in frail older adults, found a greater life-space in less frail participants and a greater effect on gait speed in more frail participants, indicating that the intervention had varying effects depending on the preconditions. A finding that should be considered in designing interventional studies. Further, the subgroup analyses revealed positive effects on participants aged 81 and older compared with younger ones for IPAQ at T_1_. An explanation might be the threshold for achieving significant improvements is lower for older adults. Those living in most rural areas (≤ 15,000 inhabitants) compared to those living in small towns (> 15,000 inhabitants) showed significant intervention effects for IPAQ and PCS12 at T_1,_ a finding suggesting that the awareness of the importance on physical component of mobility had increased and should be incorporated in future research performed in rural areas.

### Strengths and limitations

A particular strength of the study was the focus on community-dwelling older adults in a rural area, a setting that is typically excluded from other trials, which predominantly target urban populations. The primary outcome TOH was based on a GPS-derived measurement, which – in contrast to self-reported assessments – overcomes known limitations of self-reports such as overestimation [57]. Further, a small drop-out rate must be acknowledged, indicating that the intervention and study design was feasible.

However, there are several limitations associated with our study. First, as common in GPS-studies, we experienced issues leading to missing GPS data as (1) some devices did not collect GPS data properly and (2) participants forgot to take the devices with them. Both are common issues in studies based on real-life datasets and can lead to high attrition rates as observed in comparable study designs (attrition rates between 44.0% and 50.5% [57, 58]. However, missing data in our trial was at random, hence no bias is assumed. Further, we were unable to differentiate between active (e.g., walking, riding a bike) and passive (e.g., driving) transportation during the present study nor could we detect whether a participant spent time in their garden since the garden was within the home buffer zone. Both parameters would have been a valuable addition to our analyses as active transportation [59] and time spent in garden [60] have health-related benefits and increase physical activity. Another limitation to our study design may be due to the impact of the COVID-19 pandemic as our recruitment phase lasted from June 2021 till October 2022. Activities to engage for older adults were scarce during that time, not only at the beginning our recruitment phase but throughout the whole study period. Stakeholders did not want to put older adults at risk, but also older adults were reluctant to socialize which led to predominantly individual mobility behavior rather than group activities such as fall prevention and strength training groups, which also might weaken our findings. In addition to that, opportunities for public engagement like going to the museum or theatre might have been different depending on the constantly changing COVID-19 regulations. However, as we could only include participants with full vaccination status, allowing the greatest possible participation, the prospects were most likely the same. Even though we recruited mainly through invitational postal letters only participants with motivation to participate – as common in other health studies – were recruited and thus, result cannot be fully transferred to the general population. Another potential limitation arises from the inclusion of couples in our study. Although couples were assigned to the same group and we adjusted for couple effects using couple ID dummy variables, residual intra-couple influences may still remain. Shared behaviors or environmental factors within couples could have introduced additional correlations in the data that were not fully captured. Finally, our a priori calculated sample size of 254 participants given a statistical power of 95% was not reached and results may be affected by the lack of statistical power. If we would have considered a minimum power of 80% a total of 152 participants would have been needed, thus, our actual sample size would have been sufficient to detect the proposed effect.

### Implications for future research

We did not find intervention effects, which may be related to the rather active sample that we were able to recruit, and which fitted to the preventive approach that we were aiming for. Thus, reaching less active older adults in rural areas should be targeted in future studies. Further, our intervention was developed to link goal setting components with social and environmental resources, which may not work with study personnel. Although we also had staff living in the community, future studies may test interventions that draw on community workers or volunteers from the communities as done in the buddy intervention by Luger et al. [61]. Although the effects of the subgroup analysis are explorative, it hints that future studies should look more closely at age, frailty, regional and gender sensitive interventions, which can be targeted by participatory approaches. Future research can also focus on designing multidimensional mobility interventions that additionally facilitate competences like health literacy, that in turn has an effect on out-of-home mobility as Uemura et al. explored [14]. Finally, as said before, while out-of-home mobility constitutes an important outcome in older adults, more fine-grained parameters of GPS-assessed mobility need to be tested for detecting not only the temporal and spatial aspect but also the quality of mobility in terms of social, cognitive, and physical richness outside their homes.

## Conclusion

The MOBILE trial demonstrated no effect on out-of-home mobility measured by GPS-derived TOH. However, upon closer examination of subgroups, significant effects were found on secondary outcomes, including self-reported life-space mobility. These findings should be interpreted with caution and primarily highlight the need for future research. In particular, further studies are required to explore tailored interventions that specifically target distinct subgroups of older adults living in rural areas. A multidimensional approach to engaging this heterogeneous population could be more effective to improve out-of-home mobility. Additionally, incorporating novel GPS-based indicators may provide deeper insights into mobility patterns in future research.

## Supplementary Information

Supplementary Material 1. Supplementary Material MOBILE Trial.

## Data Availability

Data collected in this study will be available upon reasonable request and stored at the institutional repository after the study is completed. Data will be in aggregated form (no GPS fixes) to secure the privacy of the study participants. The architecture of the app developed specifically for this study can be accessed via the following links: https://gitlab.com/arik.grahl/mobile-project-client and https://gitlab.com/arik.grahl/mobile-project-server.
