# Supplementary material for: Out-of-home mobility enhancement by a physiotherapist-led motivational counselling intervention among rural community-dwelling older adults 75+: the MOBILE RCT
Source: BMC Geriatr. 2026 Mar 5;26:481. doi: 10.1186/s12877-026-07244-w (PMC13063870; doi:10.1186/s12877-026-07244-w)
Supplement: Supplementary file 1 — Supplementary Material 1. Supplementary Material MOBILE Trial. [file 12877_2026_7244_MOESM1_ESM.pdf]

eFigure 1: Significant intervention effect (subgroup x time) on selected outcomes and subgroups

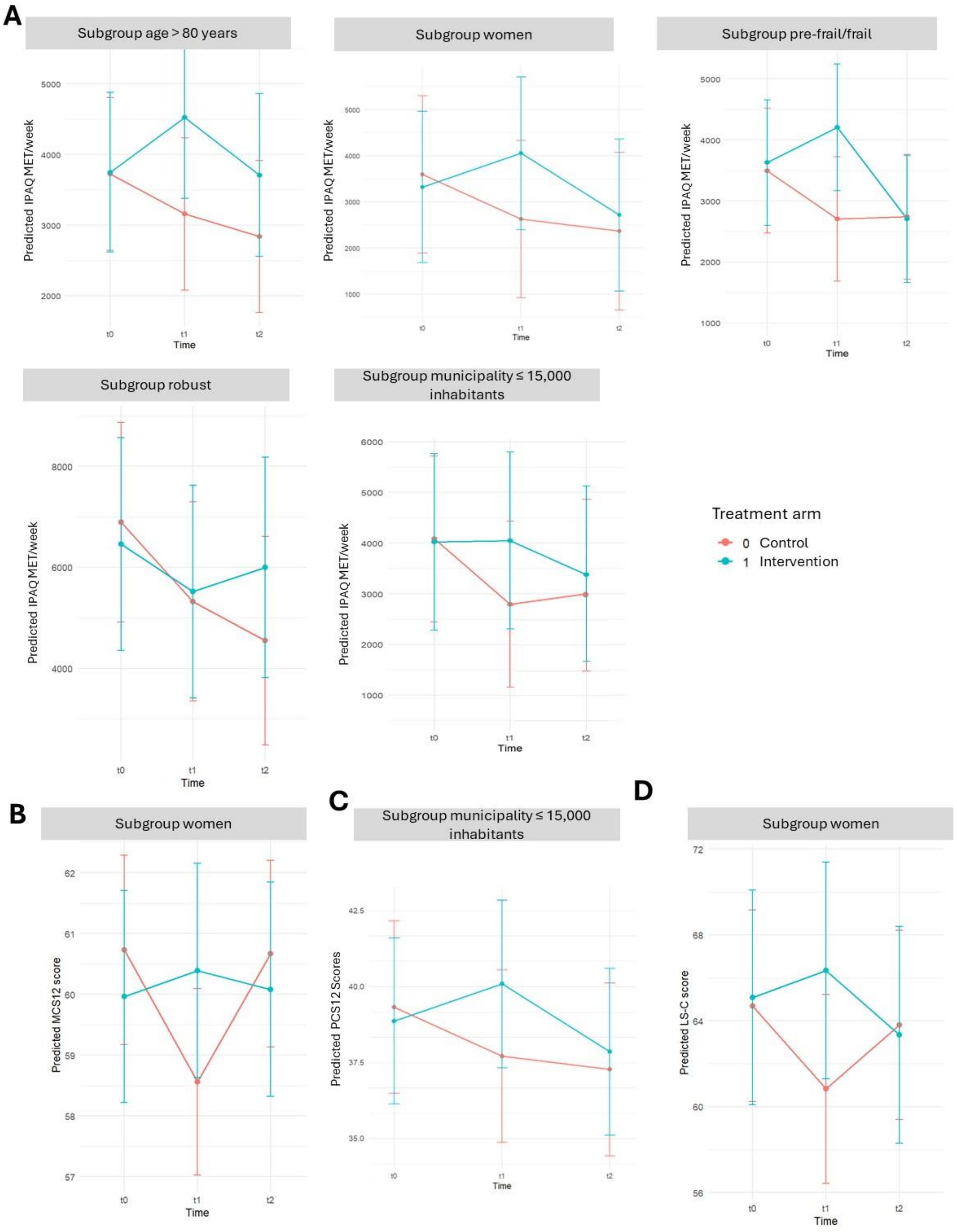

Legend: Estimated marginal means and intervention effects illustrating the relationship between intervention and control group over time (T_0 = Baseline, T_1 = 4-week follow-up, T_2 = 12-week follow-up).
Panel A: Plots for physical activity measured with self-reported IPAQ (international physical activity questionnaire) for subgroups age > 80 years (n = 104), women (n = 115), pre-frail/frail (n = 136), robust (n = 76), and most rural area (≤ 15,000 inhabitants). Panel B: Plots for mental health measured with the mental component summary (MCS12) scale of SF-12 (short form health survey) for subgroup women. Panel C: Plot for physical health measured with physical component summary (PCS12) scale of SF-12 (short form health survey) for subgroup most rural (≤ 15,000 inhabitants). Panel D: Plot for composite score (LS-C) of the Life-Space Assessment (LSA) for subgroup women. Y-axis vary due to different scaling.

eTable 1: Description of the intervention in the MOBILE trial for intervention and control group using the Template for Intervention Description and Replication (TIDieR) checklist by Hoffmann and colleagues 2014 [1]

Item	Description
1. Brief name	Intervention group: MOBILE Intervention (Motivational home visit and booster calls to enhance out-of-home mobility)<br>Control group: N/A
2. Why	Maintaining out-of-home mobility is important for older adults to continue an independent, active, and self-determined life. Out-of-home mobility defined as movement through diverse areas outside the house, can decline with increasing age as a consequence of physical, psychological and social age-related changes which in turn, can lead to adverse physical, and psychological health outcomes as well as to withdrawal from participation in social activities. In this case, strategies and plans are needed to adopt or change learned inactive behavior indoors and keep up social contact. Additionally, out-of-home mobility is a way to increase physical activity and social contact even in pre-frail or frail older adults on a daily level as the intensity and content of out-of-home mobility (e.g. participating in group exercise, walks, or being driven to visit friends) can be adjusted to the interests, competencies, and needs of the old person.<br>There is a need for interventions that foster out-of-home mobility tailored to the participant's needs and resources that can be delivered by professional and voluntary healthcare workers.
3. What: Materials	Intervention group:<br>Mobile-Intervention-App for iOS Tablets to document and visualize the first session (home visit). The app provides three screens:<br><br>1. Definition of specific personal goals for out-of-home mobility set during the counseling intervention. Chosen goals for out-of-home mobility are described by answering the four questions:<br>a) What? (Name of out-of-home activity e.g. walking, riding the bike, participation in group activities, visiting someone)<br>b) When? Define time point as precisely as possible e.g. every day at 2 pm rather than daily, every Wednesday evening rather than weekly to emphasize routines<br>c) Where? Define the place on the map or define the city/village and name the location<br>d) With whom? Name the person(s) that join the out-of-home activity or assist with the activity (e.g. Roger, even if Roger drives the car to get the participant to the place of the activity)<br>2. A person-centered social network map to visualize social contacts, personal interests, and environmental resources consisting of three circles around the target person (participant) in the center<br>a) very important, emotionally close persons, interests (the inner circle)<br>b) important persons, interests, or environmental resources (middle circle)<br>c) somewhat important persons, interests or environmental resources (outer circle)<br>Social contacts, personal interests, and environmental resources for out-of-home are additionally separated into four fields of categories:<br>a) Family<br>b) Friends and neighbors<br>c) Community<br>d) Healthcare services<br>3. Interactive map of the Havelland region with listed locations for activities (e.g. exercise groups, social meeting points) and the possibility to add new self-selected activity spots (sports club, library with social events).<br>Printer, connected to the iOS Tablet and selected goals for out-of-home mobility, as well as the person-centered social network map can be printed out for visualizing and documentation for the participant.<br>Flyer and an information sheet with information about the general aims of the study, the process of the intervention and control condition, data protection, and contact information<br>Control group:<br>8-page booklet containing general information about the positive effects of out-of-home mobility, tips for a more active lifestyle, and regional hints for engaging in activities.

	Flyer and an information sheet with information about the general aims of the study, the process of the intervention and control condition, data protection, and contact information All materials are written in German and are available upon request.
4. What: Procedures	Intervention group:<br>There are three motivational counseling sessions.<br>Session 1: First, the intervention and the materials were introduced and explained and benefits of out-of-home mobility were demonstrated. Relatives who wanted to be around were allowed to, but were asked to stay uninvolved, since the focus should remain on the participant. The content of the first session was structured by the three screens of the Mobile-Intervention-App. First, the personal network was filled out together and thus, highlighting the existing social network and possibly unused resources. The participants chose how to fill in the map (real names and/or dummies) and no target number was predetermined. The social network map is person-centered and has three circles representing very important, important, and less important person/institutions clustered into four domains (family, friends and neighbors, community, healthcare services). After filling out the social network map, individual mobility goals were chosen that could either be routine goals (e.g. walking the dog every morning) or specials mobility goals (e.g. going to the museum on the weekend). By setting the goals a clear plan was made including – amongst other - the questions "when", "where" and "with whom" and behavioral change techniques were applied [2] , that can be retraced in detail in the study protocol[3].<br>The participants either chose goals by themselves or could take a look at the interactive map, where activities to engage with in the near environment are presented. The session took around 90-120min.<br>Session 2 and 3: There are two booster calls (4 and 8 weeks after the first session). The foundation of these sessions are the defined mobility goals that have been established in the first interview and were printed out. The network map and the goals are reviewed and discussed whether it was well chosen or if unexpected barriers occurred. Goal adaption was then individually and flexibly adjusted to personal circumstances. The sessions not only focused on barriers, but also affirmed and encouraged, especially when the chosen goal was to maintain the current mobility status. A documentation of the goal adaption session were done by the physiotherapists and sent to person' home, when whished for. Time for the telephone session differed between 15 and 45 min.<br>Control group: N/A
5. Who provided	Intervention group:<br>Trained physiotherapists that received a manual about the intervention, goal-setting techniques and behavior-change techniques.<br>Physical therapists were trained in using the MOBILE- Intervention iOS App.<br>Physical therapists were also trained in establishing first contact with participants at their homes and communicating with authenticity and an appreciative attitude during all sessions.<br>Control group:<br>The information booklet was sent to participants of the control group after randomization and conduction of the baseline questionnaire and seven days of mobility assessment via GPS
6. How	The intervention was delivered as a home visit (session 1) and booster phone calls (session 2 and 3).
7. Where	Study center was in Berlin, intervention was delivered in the region Havelland (ca. 30km west of Berlin)
8. When and how much	Intervention group:<br>1 home visit (90-120 min), 2 booster phone calls (15-45 min, 4 and 8 weeks after the first session).<br>Control group: N/A
9. Tailoring	Intervention group:<br>Goals were tailored for each participant individually according to abilities, resources, and barriers. To The booster sessions acted as a further possibility to tailor to new circumstances or personal capacities.<br>Control group: N/A
10. Modifications	Intervention group:<br>Couples are also enrolled in the study and intervention was priory planned to be separated for each partner. However, after the first sessions with some couples it became clear, that

	in some cases it makes sense to deliver the intervention together (as some couples are engaging in the same activities). Thus, the manual was changed and each couple was asked whether the intervention should be delivered together or individually.<br>Control group: N/A
11. How well: Planned	Intervention group:<br>Adherence to the intervention (i.e. taking part in the sessions) was assessed in a database by the physiotherapist. Adherence to self-chosen mobility goals was documented during the booster calls.<br>Control group: N/A
12. How well: Actual	Intervention group:<br>109 participants were randomized to the intervention group and all took part in session 1. 107 participants took part in session 2 and 103 in session 3, thus adherence rate to intervention was 94.5 %. Adherence to the self-chosen mobility goals was documented in the booster calls, but was not standardized, thus no rate can be reported.<br>Control group: N/A

1. Hoffmann TC, Glasziou PP, Boutron I, et al. Better reporting of interventions: Template for intervention description and replication (TIDieR) checklist and guide. *BMJ*. 2014;348(March):1-12. doi:10.1136/bmj.g1687
2. Michie S, Richardson M, Johnston M, et al. The behavior change technique taxonomy (v1) of 93 hierarchically clustered techniques: Building an international consensus for the reporting of behavior change interventions. *Ann Behav Med*. 2013;46(1):81-95. doi:10.1007/s12160-013-9486-6
3. Haeger C, Mümken SA, O‘Sullivan JL, et al. Mobility enhancement among older adults 75 + in rural areas: Study protocol of the MOBILE randomized controlled trial. *BMC Geriatr*. 2022;22(1):1-11. doi:10.1186/s12877-021-02739-0

eTable 2: Baseline characteristics of completers and dropouts of the MOBILE trial (N = 212)

Categorical variables	Completer (n = 198) n (%)	Dropouts (n = 14) n (%)	*P* - Value
Gender, women	109 (55.1)	10 (71.4)	.233
Population size of municipality, ≤ 15,000 inhabitants	105 (53.0)	4 (28.6)	.080
Education			
Basic	3 (1.5)	2 (14.3)	< .001
Vocational	76 (38.4)	9 (64.3)	
Degree	119 (60.1)	3 (21.4)	
Level of care			
No level of care	159 (80.3)	9 (64.3)	.091
Level of care 1	10 (5.1)	3 (21.4)	
Level of care 2	25 (12.6)	2 (14.3)	
Level of care 3	4 (2.0)	/	
Living arrangements			
Living alone	76 (38.4)	9 (64.3)	.283
Not living alone	122 (61.2)	5 (35.7)	
Use of assistive devices			
Walking aid	44 (22.2)	8 (57.1)	.003
Hearing aid	72 (36.4)	3 (21.4)	.259
Visual aid	194 (98.0)	14 (100.0)	.591
Smartphone	172 (86.9)	11 (78.6)	.383
Frailty			
Robust	71 (36.4)	2 (14.3)	.135
Pre-frail	106 (54.4)	9 (64.3)	
Frail	18 (9.2)	3 (21.4)	
Physical activity (IPAQ)			
low	19 (9.6)	1 (7.2)	.829
Moderate	31 (15.7)	3 (21.4)	
high	148 (74.7)	10 (71.4)	
Continuous variables	Mean (SD)	Mean (SD)	
Age	81.4 (4.0)	83.3 (4.8)	.383
Mobility			
TOH[1]	313.9 (190.2)	395.7 (266.7)	.052
CHull			
LS-C	78.5 (19.6)	64.1 (24.7)	.159
Physical activity			
MET-minutes/week	5019.80 (2989.32)	4241.93 (2167.70)	.225
Depressive symptoms			
GDS-12-R	0.95 (1.5)	1.57 (1.8)	.247
Health status SF-12			
PCS12	44.4 (11.1)	38.1 (13.1)	.193
MCS12	58.3 (4.9)	59.2 (5.3)	.845

[1] Data of 187 completers and 13 dropouts.

Note: Values are presented frequencies for categorical and in means and standard deviation (SD) for continuous variables. P-values are reported as unpaired t-tests for continuous and Pearson's $\chi 2$ test for categorical data. SD = Standard Deviation, TOH = Time-out-of-home, CHull = Convex Hull (= GPS-Based Life-Space parameter), LS-C = Composite Score of the Life-Space Assessment, IPAQ = International Physical Activity Questionnaire, MET = metabolic equivalent of an activity, GDS-12R = Geriatric Depression Scale, Residential, SF-12 = Short Form Health Survey, PCS12 = Physical Component Summary Scale of SF-12, MCS12 = Mental Component Summary Scale of SF-12

eTable 3: Baseline characteristics of MOBILE trial participants (N = 212) differentiated for subgroup gender

	Men (n = 93)		Women (n = 119)	
	IG (n = 48)<br>n (%)	CG (n = 45)<br>n (%)	IG (n = 60)	CG (n = 59)
Gender, women	-	-	-	-
Age, mean (SD)	80.1 (3.1)	82.0 (4.1)	82.0	81.8 (4.6)
Population size of municipality, ≤ 15,000 inhabitants	31 (64.6)	19 (42.2)	28 (46.7)	31 (52.5)
Education				
Basic	1 (2.1)	1 (2.2)	2 (3.3)	1 (1.7)
Vocational	13 (27.1)	16 (35.6)	25 (41.7)	31 (52.5)
Degree	34 (70.8)	28 (62.2)	33 (55.0)	27 (45.8)
Level of care				
No level of care	42 (87.5)	42 (93.3)	46 (76.7)	38 (64.4)
Level of care 1	0 (0)	0 (0)	5 (8.3)	8 (13.6)
Level of care 2	6 (12.5)	2 (4.4)	8 (13.3)	11 (18.6)
Level of care 3	0 (0)	1 (2.2)	1 (1.7)	2 (3.4)
Living arrangements				
Living alone	10 (20.8)	7 (15.6)	31 (51.7)	37 (62.7)
Not living alone	38 (79.2)	38 (84.4)	29 (48.3)	22 (37.3)
Use of assistive devices				
Walking aid	6 (12.5)	9 (20.0)	18 (30.0)	19 (32.2)
Hearing aid	16 (33.3)	18 (40.0)	20 (33.3)	21 (35.6)
Visual aid	47 (97.9)	43 (95.6)		58 (98.3)
Smartphone	44 (91.7)	38 (84.4)		50 (84.7)
Mobility				
TOH, mean (SD) (n = 201)	294.1 (139.4)	347.6 (192.8)	332.2 (240.4)	302.1 (184.4)
CHull, mean (SD) (n = 201)	38.9 (51.7)	74.7 (258.9)	31.1 (64.8)	31.0 (63.2)
LS-C, mean (SD)	85.5 (18.5)	80.7 (17.1)	74.9 (22.3)	71.5 (19.3)
Physical Activity (IPAQ)				
low	5 (10.4)	2 (4.4)	3 (5.0)	10 (16.9)
moderate	5 (10.4)	9 (20.0)	13 (21.7)	7 (11.9)
high	38 (79.2)	34 (75.6)	44 (73.3)	42 (71.2)
MET-minutes/Week, Mean (SD)	5547.6 (3373.4)	5150.8 (2945.1)	4692.0 (2598.8)	4639.2 (2894.2)
Depressive symptoms				
GDS-12R, mean (SD)	0.8 (1.0)	0.8 (1.5)	0.9 (1.6)	1.4 (1.9)
Health Status SF-12				
PCS12, mean (SD)	45.5 (58.6)	48.0 (8.7)	41.4 (12.8)	41.5 (11.2)
MCS12, mean (SD)	58.6 (5.1)	57.7 (3.9)	59.1 (4.7)	57.9 (5.8)
Frailty (n=209)				
Robust	18 (37.5)	15 (33.3)	25 (41.7)	15 (25.9)
Pre-frail	27 (56.3)	27 (62.8)	28 (46.7)	33 (56.9)
Frail	3 (6.3)	1 (2.3)	7 (11.7)	10 (17.2)

Values are presented in means and standard deviation (SD) and in frequencies for categorical data. Note: IG = Intervention Group, CG = Control Group, SD = Standard Deviation, TOH = Time-out-of-home, CHull = Convex Hull (= GPS-Based Life-Space parameter), LS-C = Composite Score of the UAB Life-Space Assessment), IPAQ = International Physical Activity Questionnaire, MET = metabolic equivalent of an activity, GDS-12R = Geriatric Depression Scale, Residential, SF-12 = Short Form Health Survey, PCS12 = Physical Component Summary Scale of SF-12, MCS = Mental Component Summary Scale of SF-12

eTable 4: Baseline characteristics of MOBILE trial participants (N = 212) differentiated by subgroup frailty status

	Pre-frail or frail (n = 136)		Robust (n = 77)	
	IG (n = 65)<br>n (%)	CG (n = 71)<br>n (%)	IG (n = 43)<br>n (%)	CG (n = 33)<br>n (%)
Gender, women	35 (53.8)	43 (60.6)	25 (58.1)	16 (48.4)
Age, mean (SD)	82.4 (4.0)	83.2 (4.4)	79.4 (2.5)	79.1 (2.7)
Population size of municipality, ≤ 15,000 inhabitants	35 (53.8)	33 (46.5)	24 (55.8)	17 (51.5)
Education				
Basic	2 (3.1)	2 (2.8)	1 (2.3)	0 (0)
Vocational	25 (38.5)	35 (49.3)	13 (30.2)	12 (36.4)
Degree	38 (58.5)	34 (47.9)	29 (67.4)	21 (63.6)
Level of care				
No level of care	45 (69.2)	47 (66.2)	43 (100)	33 (100)
Level of care 1	5 (7.7)	8 (11.3)	0 (0)	0 (0)
Level of care 2	14 (21.5)	13 (18.3)	0 (0)	0 (0)
Level of care 3	1 (1.5)	3 (4.2)	0 (0)	0 (0)
Living arrangements				
Living alone	29 (44.6)	36 (50.7)	12 (27.9)	8 (24.2)
Not living alone	36 (56.4)	34 (49.3)	31 (72.1)	25 (75.7)
Use of assistive devices				
Walking aid	24 (36.9)	27 (38.0)	0 (0)	1 (3.0)
Hearing aid	23 (35.4)	30 (42.3)	13 (30.2)	9 (27.3)
Visual aid	65 (100)	69 (97.2)	43 (97.7)	32 (97.0)
Smartphone	53 (81.5)	59 (83.1)	42 (97.7)	29 (87.9)
Mobility				
TOH, mean (SD) (n = 201)	330.7 (253.4)	305.3 (197.9)	296.2 (102.5)	359.8 (163.1)
CHull, mean (SD) (n = 201)	30.6 (66.3)	57.0 (210.7)	39.7 (48.7)	36.1 (72.8)
LS-C, mean (SD)	73.5 (21.8)	71.5 (19.6)	89.0 (16.8)	84.0 (14.3)
Physical Activity (IPAQ)				
low	8 (12.3)	12 (16.9)	0 (0)	0 (0)
moderate	12 (18.5)	13 (18.3)	6 (14.0)	3 (9.1)
high	45 (69.2)	46 (64.8)	37 (86.0)	30 (90.9)
MET-minutes/Week, Mean (SD)	4366.5 (3076.8)	4137.2 (2659.6)	6139.2 (2514.9)	6416.8 (2861.2)
Depressive symptoms				
GDS-12R, mean (SD)	1.0 (1.5)	1.5 (1.9)	0.5 (1.2)	0.5 (0.9)
Health Status SF-12				
PCS12, mean (SD)	40.0 (13.0)	42.6 (11.5)	47.7 (7.4)	49.9 (7.2)
MCS12, mean (SD)	59.3 (5.4)	57.9 (5.5)	58.4 (3.7)	57.8 (3.8)
Frailty (n=209)				
Robust	0 (0)	0 (0)	43 (100)	33 (100)
Pre-frail	55 (84.6)	60 (84.5)	0 (0)	0 (0)
Frail	10 (15.4)	11 (15.5)	0 (0)	0 (0)

Values are presented in means and standard deviation (SD) and in frequencies for categorical data. Note: IG = Intervention Group, CG = Control Group, SD = Standard Deviation, TOH = Time-out-of-home, CHull = Convex Hull (= GPS-Based Life-Space parameter), LS-C = Composite Score of the UAB Life-Space Assessment), IPAQ = International Physical Activity Questionnaire, MET = metabolic equivalent of an activity, GDS-12R = Geriatric Depression Scale, Residential, SF-12 = Short Form Health Survey, PCS12 = Physical Component Summary Scale of SF-12, MCS = Mental Component Summary Scale of SF-12

eTable 5: Baseline characteristics of MOBILE trial participants (N = 212) differentiated by subgroup age

	Age 75-80 (n = 104)		Age ≥ 81 (n = 108)	
	IG (n = 55)<br>n (%)	CG (n = 49)<br>n (%)	IG (n = 53)<br>n (%)	CG (n = 55)<br>n (%)
Gender, women	30 (54.5)	19 (38.8)	18 (34.0)	26 (47.3)
Age, mean (SD)	78.2 (1,2)	78.2 (1.4)	84.2 (3.0)	85.35 (3.3)
Population size of municipality, ≤ 15,000 inhabitants	37 (67.3)	26 (53.1)	22 (41.5)	24 (43.6) (=2)
Education				
Basic	--	--	3 (5.7)	2 (3.6)
Vocational	17 (30.9)	19 (38.8)	21 (39.6)	28 (50.9)
Degree	38 (69.1)	30 (61.2)	29 (54.7)	25 (45.5)
Level of care				
No level of care	51 (92.7)	44 (89.8)	37 (69.8)	36 (65.5)
Level of care 1	--	1 (2)	5 (9.4)	7 (12.7)
Level of care 2	4 (7.3)	1 (2)	10 (18.9)	12 (21.8)
Level of care 3	--	3 (6.1)	1 (1.9)	--
Living arrangements				
Living alone	12 (21.8)	16 (32.7)	29 (54.7)	28 (50.9)
Not living alone	43 (78.2)	33 (67.3)	24 (45.3)	27 (49.1)
Use of assistive devices				
Walking aid	5 (9.1)	5 (10.2)	19 (35.8)	23 (41.8)
Hearing aid	16 (29.1)	12 (24.5)	20 (37.7)	27 (49.1)
Visual aid	54 (98.2)	48 (98.0)	53 (100.0)	53 (96.4)
Smartphone	52 (94.5)	46 (93.9)	43 (81.1)	42 (76.4)
Mobility				
TOH, mean (SD) (n = 201)	318.5 (200.8)	395.3 (207.8)	314.1 (210.5)	259.0 (144.4)
CHull, mean (SD) (n = 201)	43.2 (73.6)	1638.9 (245.0)	24.9 (38.0)	30.0 (87.2)
LS-C, mean (SD)	87.9 (19.2)	82.5 (16.3)	71.1 (20.1)	69.2 (19.0)
Physical Activity (IPAQ)				
low	2 (3.6)	2 (4.1)	6 (11.3)	10 (18.2)
moderate	5 (9.1)	6 (12.2)	13 (24.5)	10 (18.2)
high	48 (87.3)	41 (83.7)	34 (64.2)	34 (63.6)
MET-minutes/Week, Mean (SD)	6288.7 (3013.5)	6050.6 (2939.7)	3810.1 (2388.4)	3800.3 (2465.1)
Depressive symptoms				
GDS-12R, mean (SD)	0.6 (1.1)	0.9 (1.7)	1.2 (1.6)	1.4 (1.8)
Health Status SF-12				
PCS12, mean (SD)	46.1 (9.9)	47.0 (9.1)	40.1 (12.6)	42.7 (11.9)
MCS12, mean (SD)	58.6 (4.5)	56.8 (5.3)	59.2 (5.2)	58.7 (4.6)
Frailty (n=209)				
Robust	32 (58.2)	25 (53.2)	11 (20.8)	5 (9.2)
Pre-frail	18 (32.7)	22 (46.8)	37 (69.8)	38 (70.4)
Frail	5 ((9.1)	--	5 (9.4)	11 (20.4)

Values are presented in means and standard deviation (SD) and in frequencies for categorical data. Note: IG = Intervention Group, CG = Control Group, SD = Standard Deviation, TOH = Time-out-of-home, CHull = Convex Hull (= GPS-Based Life-Space parameter), LS-C = Composite Score of the UAB Life-Space Assessment), IPAQ = International Physical Activity Questionnaire, MET = metabolic equivalent of an activity, GDS-12R = Geriatric Depression Scale, Residential, SF-12 = Short Form Health Survey, PCS12 = Physical Component Summary Scale of SF-12, MCS = Mental Component Summary Scale of SF-12

eTable 6: Baseline characteristics of MOBILE trial participants (N = 212) differentiated for subgroup by population size of municipality

	Population size of municipality > 15,000 inhabitants (n = 103)		Population size of municipality ≤ 15,000 inhabitants (n = 109)	
	IG (n = 49)<br>n (%)	CG (n = 54)<br>n (%)	IG (n = 59)	CG (n = 50)
Gender, women	17 (34.7)	26 (48.1)	31 (52.5)	19 (38.0)
Age, mean (SD)	82.2 (4.0)	82.6 (4.6)	80.3 (3.3)	81.2 (4.09
Population size of municipality, ≤ 15,000 inhabitants	-	-	-	-
Education				
Basic	1 (2.0)	1 (1.9)	2 (3.4)	1 (2.0)
Vocational	20 (40.8)	25 (46.3)	18 (30.5)	22 (4.0)
Degree	28 (57.1)	28 (51.9)	39 (66.1)	27 (54.0)
Level of care				
No level of care	38 (77.6)	42 (77.8)	50 (84.7)	38 (76.0)
Level of care 1	3 (6.1)	3 (5.6)	2 (3.4)	5 (10.0)
Level of care 2	7 (14.3)	8 (14.8)	7 (11.9)	5 (10.0)
Level of care 3	1 (2.0)	1 (1.9)	0 (0)	2 (4.0)
Living arrangements				
Living alone	23 (46.9)	23 (42.6)	18 (30.5)	21 (42.0)
Not living alone	26 (53.0)	29 (53.7)	41 (69.5)	29 (58.0)
Use of assistive devices				
Walking aid	15 (30.6))	14 (25.9)	9 (15.3)	14 (28.0)
Hearing aid	19 (38.8)	21 (38.9)	17 (28.8)	18 (36.0)
Visual aid	49 (100)	53 (98.1)	58 (98.3)	48 (96.0)
Smartphone	43 (87.8)	46 (85.2)	52 (88.1)	42 (84.0)
Mobility				
TOH, mean (SD) (n = 201)	380.0 (267.9)	349.5 (210.9)	263.3 (106.2)	295.7 (161.5)
CHull, mean (SD) (n = 201)	34.8 (72.1)	73.5 (249.7)	33.9 (47.1)	28.9 (46.3)
LS-C, mean (SD)	75.7 (21.0)	74.8 (20.0)	82.9 (21.0)	76.1 (17.8)
Physical Activity (IPAQ)				
low	2 (4.1)	8 (14.8)	6 (10.2)	4 (8.0)
moderate	10 (20.4)	8 (14.8)	8 (13.6)	8 (16.0)
high	37 (75.5)	38 (70.4)	45 (76.3)	38 (76.0)
MET-minutes/Week, Mean (SD)	4781.2 (2720.9)	4599.0 (2777.2)	5314.0 (3189.0)	5142.9 (3056.3)
Depressive symptoms				
GDS-12R, mean (SD)	0.9 (1.3)	0.8 (1.2)	0.7 (1.4)	1.4 (2.1)
Health Status SF-12				
PCS12, mean (SD)	42.4 (12.3)	45.5 (11.4)	43.8 (11.2)	43.9 (10.3)
MCS12, mean (SD)	58.3 (5.0)	58.4 (4.7)	59.4 (4.6)	57.2 (5.3)
Frailty (n=209)				
Robust	19 (38.8)	16 (29.6)	24 (40.7)	14 (29.8)
Pre-frail	24 (49.0)	32 (59.3)	31 (52.5)	28 (59.6)
Frail	6 (12.2)	6 (11.1)	4 (6.8)	47 (10.6)

Values are presented in means and standard deviation (SD) and in frequencies for categorical data. Note: IG = Intervention Group, CG = Control Group, SD = Standard Deviation, TOH = Time-out-of-home, CHull = Convex Hull (= GPS-Based Life-Space parameter), LS-C = Composite Score of the UAB Life-Space Assessment), IPAQ = International Physical Activity Questionnaire, MET = metabolic equivalent of an activity, GDS-12R = Geriatric Depression Scale, Residential, SF-12 = Short Form Health Survey, PCS12 = Physical Component Summary Scale of SF-12, MCS = Mental Component Summary Scale of SF-12
